# Supplementary material for: Translating a Thin-Film Rehydration Method to Microfluidics for the Preparation of a SARS-CoV-2 DNA Vaccine: When Manufacturing Method Matters
Source: Pharmaceutics. 2022 Jul 7;14(7):1427. doi: 10.3390/pharmaceutics14071427 (PMC9316859; doi:10.3390/pharmaceutics14071427)
Supplement: Supplementary file 1 [file pharmaceutics-14-01427-s001.zip › pharmaceutics-1762758-supplementary.pdf]

Supplementary figure S1:

WST-1 assays on RAW 264.7 cells after 24h incubation with lipoplexes manufactured by microfluidics

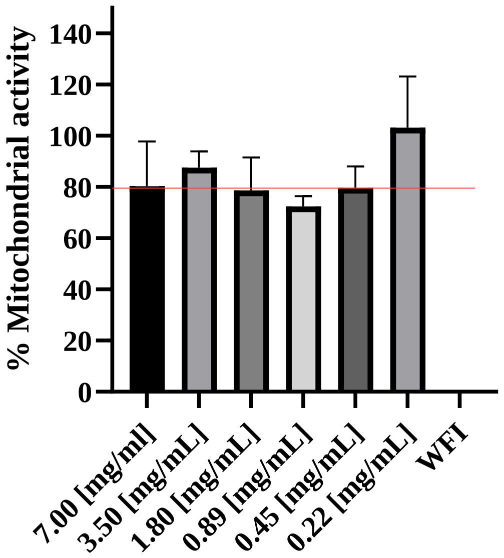

Fig S1. Toxicity of different concentrations of blank DOTAP 4 liposomes manufactured by microfluidics (MF) method liposomes on RAW 264.7 cells, incubated on cells for 24 h. Data were normalized to non-treated cell results.
